# Correlations between tear lymphocytotoxin-a (LT-α) level and disease severity in adult keratoconus

**DOI:** 10.3389/fmed.2025.1591159

**Published:** 2025-06-06

**Authors:** Xiuxian Qin, Shunrong Luo, Xie Fang, Xianwen Xiao, Zhiwen Xie, Xumin Shang, Juan Yang, Xi Lan, Huping Wu, Zhirong Lin

**Affiliations:** ^1^Xiamen Eye Center and Eye Institute of Xiamen University, Xiamen, China; ^2^Guangzhou Huangpu Aier Eye Hospital, Guangzhou, China; ^3^Xiamen Clinical Research Center for Eye Diseases, Xiamen, China; ^4^Xiamen Key Laboratory of Ophthalmology, Xiamen, China; ^5^Fujian Key Laboratory of Corneal & Ocular Surface Diseases, Xiamen, China; ^6^Xiamen Key Laboratory of Corneal & Ocular Surface Diseases, Xiamen, China; ^7^Translational Medicine Institute of Xiamen Eye Center of Xiamen University, Xiamen, China

**Keywords:** keratoconus, lymphotoxin-α (LT-α), tear film, corneal tomography, inflammation, point-of-care test

## Abstract

**Purpose:**

This study aimed to investigate tear lymphocytotoxin-*α* (LT-α) levels in adult keratoconus patients and identify the correlations between LT-α and the disease severity of keratoconus.

**Methods:**

In this cross-sectional study, tear samples from 100 keratoconus patients (KC group) and 67 healthy controls (HC group) were collected. LT-α levels were tested immediately after collection using a commercially available point-of-care test (POCT). Visual acuity, corneal tomography, anterior segment optical coherence tomography (AS-OCT), tear film parameters, including non-invasive break-up time (NI-BUT), tear meniscus height (TMH), lipid layer thickness (LLT), and meibomian glands, were also tested. Corneal topography values (K1, K2, K_max,_ and astigmatism) were derived from the tomography data.

**Results:**

The tear LT-α levels in the HC group were significantly higher than those in the KC group (*p* = 0.038). The NI-BUT, TMH, and LLT were significantly lower, while the meiboscore was higher in the KC group than in the HC group (*p* < 0.001). In the KC group, patients were further divided into preclinical stage, initial stage, and complete stage groups. Significant differences in tear LT-α levels were found among the three subgroups. Within the KC group, LT-α levels were significantly correlated with various parameters, including thinnest corneal thickness, best-corrected visual acuity (BCVA) (LogMAR), K1, K2, K_max_, and astigmatism values. However, no significant correlation was observed between the tear LT-α level and tear film parameters. Within the control group, no significant correlation was identified between the tear LT-α level and corneal topography or tear film parameters.

**Conclusion:**

Our data demonstrated an association between tear LT-α levels and the severity of keratoconus, implying that immune-related factors may be involved in the pathogenesis and progression of the disease.

## Introduction

Keratoconus is a progressive corneal disorder characterized by corneal thinning, ectasia, and protrusion of the central cornea, resulting in irregular astigmatism and visual impairment (1). Although the course of the disease is relatively stable in most cases, a subset of patients progresses to advanced stages requiring corneal transplantation as an intervention to restore vision. Emerging evidence suggests a significant association between keratoconus and dry eye syndrome, with clinical studies demonstrating more severe ocular surface symptoms and greater tear film instability in keratoconus patients compared to healthy controls (2). Although the exact pathogenesis of keratoconus is still not fully understood, current research suggests that inflammatory mediators and lymphocytes are involved in the disease process (3). Inflammatory cytokines modulate various biochemical pathways contributing to corneal ectasia (4). Notably, Emine et al. have provided substantial evidence supporting that lymphocytes play a crucial role in the development and progression of keratitis pathology (5).

Lymphotoxin-alpha (LT-α), a pivotal cytokine within the tumor necrosis factor (TNF) superfamily, is encoded within the major histocompatibility complex (MHC) class III genomic region (6). This multipotent cytokine is expressed by a variety of immune cell populations, including CD4 + T helper cells, CD8 + cytotoxic T cells, natural killer (NK) cells, B lymphocytes, and macrophages, each of which has a different subunit configuration (7). Many studies have confirmed that LT-α is a multifunctional immunomodulator and plays a crucial role in the proprioceptive development and functional regulation of the immune system (8). Its biological significance extends to lymphoid tissue organogenesis, immune cell differentiation, and inflammatory responses, making it a key mediator in immune system balance and pathogenesis (9–11).

Chiang et al. demonstrated that LT-α expression was significantly upregulated in activated human donor lymphocytes (12). It is notable that clinical observations showed a significant reduction in the percentage of lymphocytes and absolute lymphocyte counts in patients with progressive keratoconus compared to healthy controls and patients with non-progressive keratoconus (5). Despite these findings, the precise relationship between LT-α and the pathogenesis of keratoconus remains unclear. To elucidate the potential relationship between LT-α levels, keratoconus progression, and tear film stability, we conducted a comprehensive study involving the KC and HC groups. All participants underwent extensive ocular surface evaluation, including quantification of tear LT- *α* levels, medical optometry, non-invasive break-up time (NI-BUT), tear meniscus height (TMH), lipid layer thickness (LLT), meibomian gland dysfunction (MGD) assessment (using infrared imaging), anterior segment optical coherence tomography (AS-OCT), and corneal topography. This study aimed to investigate the correlation between LT-α levels and the occurrence and development of cone cornea, as well as to analyze the relationship between tear fluid LT-α concentration and tear film stability parameters in patients with keratoconus.

## Materials and methods

### Ethics and clinical subjects

The study was conducted in strict accordance with the ethical principles outlined in the Declaration of Helsinki. Before participation, written informed consent was obtained from all subjects after completely explaining the study protocol, potential risks, and expected benefits. All research procedures were conducted under the continuous supervision and monitoring of the Medical Ethics Committee to ensure compliance with established ethical standards and regulatory requirements.

The study cohort comprised 100 patients with KC and 67 age-matched HC. The inclusion criteria for the KC group were as follows: (1) a diagnosis of primary keratoconus according to the Chinese Expert Consensus on Diagnosis and Treatment of Keratoconus (2019), including latent, incipient, and progressive stages; (2) characteristic corneal topographic findings: central corneal refractive power >46.40 diopters(D), inferior–superior (I–S) value of >1.26 D, and interocular corneal refractive power difference of >0.92 D; (3) BCVA of ≥0.1; and (4) age ≥20 years.

The exclusion criteria for the KC group included the following: (1) the presence of total corneal scarring with BCVA <0.1, which meets the diagnostic criteria for keratoconus scarring stage; (2) use of ocular or systemic medications in the past 6 months; (3) history of ocular surgery, history of ocular trauma, or coexisting ocular pathology (including, but not limited to, ocular manifestations of glaucoma, retinopathy, or systemic autoimmune disorders); (4) long-term use of ocular medications; (5) systemic comorbidities such as diabetes mellitus, hypertension, and cardiovascular disease; (6) neuropathic pain disorders (e.g., trigeminal neuralgia); (7) current pregnancy; (8) use of psychotropic medications within the last 2 years; and (9) failure to meet follow-up requirements.

The clinical staging of keratoconus was classified into preclinical, initial, and complete stages according to the Chinese Expert Consensus on Diagnosis and Treatment of Keratoconus (2019) (13) (Table 1).

**Table 1:** Clinical staging and staging criteria for keratoconus.

Clinical stages	Staging criteria
Preclinical stage	Diagnosis of keratoconus in one eye, unaffected vision in the other eye, and normal corneal topography results
Initial stage	Confirmed keratoconus with BCVA ≥ 0.8
Complete stage	Confirmed diagnosis of keratoconus, BCVA<0.8, typical clinical signs on slit lamp examination, and markedly abnormal corneal topography findings.

The inclusion criteria for the HC group: (1) absence of clinical signs or symptoms of keratoconus or other ocular pathologies; (2) normal corneal topographic parameters: central corneal refractive power <46.00 D, inferior–superior (I–S) value <1.26 D, interocular corneal refractive power difference <0.92 D; (3) normal tear film parameters: NI-BUT≥10 s, TMH ≥ 0.4 mm, LLT ≥ 60 nm; (4) BCVA ≥0.8; and (5) age ≥20 years.

The exclusion criteria for the HC group included all criteria applied to the KC group, in addition to the following conditions: (1) high myopia (spherical equivalent ≤ −6.00 D); (2) corneal astigmatism >1.50 D; and (3) family history of keratoconus in first-degree relatives.

### Visual acuity test

A visual acuity test was conducted using internationally standardized optometric equipment. The following parameters were recorded: BCVA, spherical power, and cylindrical power.

### LT-α analysis in the tears

**Figure 1:**
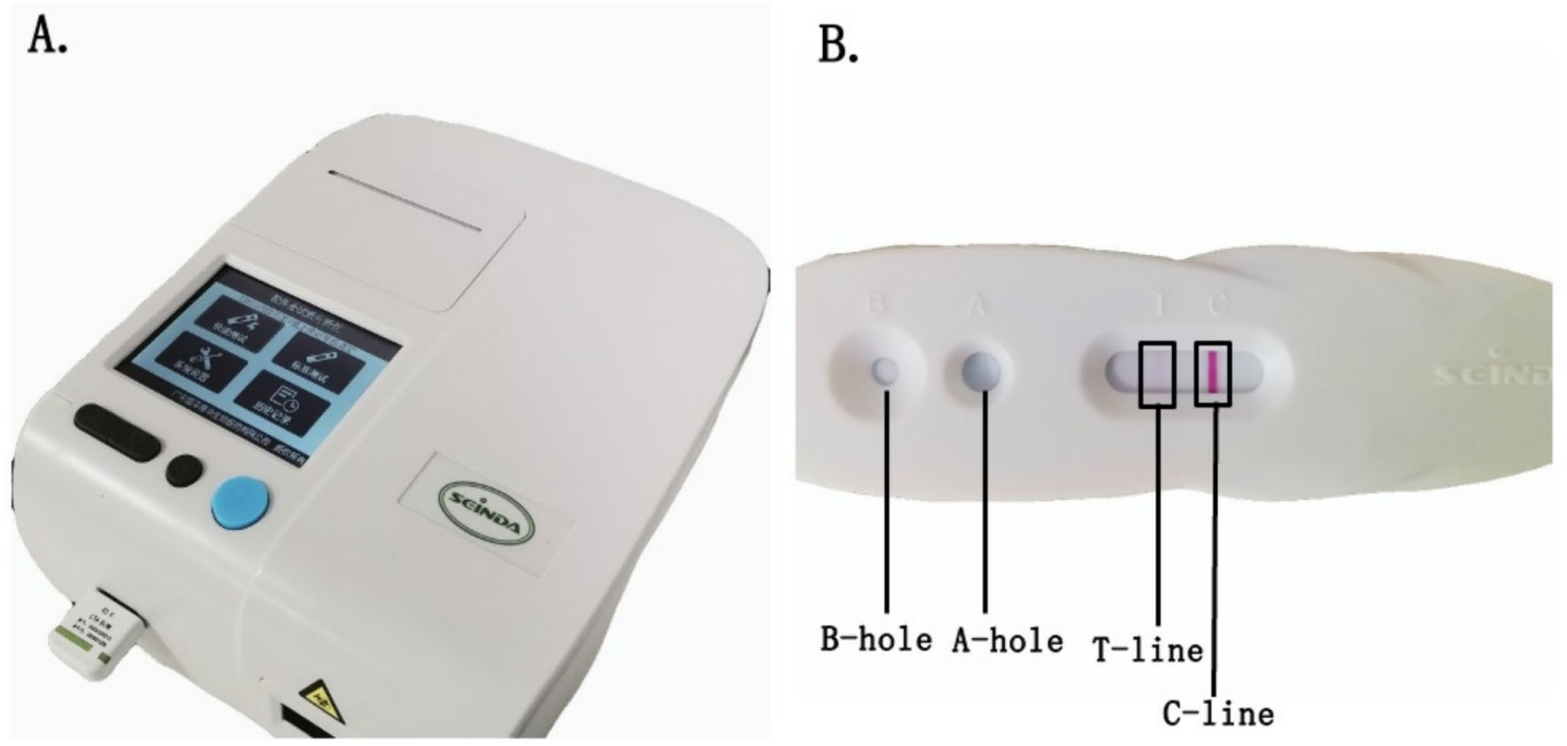
LT-α analysis. The LT-α analysis system comprises a quantitative analyzer **(A)**, NaCl-based detection solution, and a reagent card (**B**, Model S02A) containing nanomicrosphere-labeled anti-LT-α antibodies. During testing, the tear sample is added to well A of the reagent card, followed by approximately three drops of detection solution in well B. The LT-α in the sample binds to the labeled antibodies and migrates to the test zone (T-line), producing a visible colorimetric reaction. In contrast, unbound antibodies move to the control line (C-line) as a quality control. The T-line’s color intensity directly correlates with LT-α concentration, and the quantitative analyzer automatically interprets the results. This system offers rapid, sensitive, and user-friendly POCT for LT-α detection in tear samples.

The tear lymphocytotoxin *in vitro* rapid immunoassay system comprises three components: a micro tear collector, an LT-α reagent card, and a quantitative analyzer. This system provides a quantitative, rapid, and user-friendly analysis. The procedure was performed as follows (14): A disposable capillary tear collector was used to collect diluted tear fluid under slit-lamp illumination. It should be noted that the tear collection followed standardized protocols to minimize pre-analytical variability. The collected tear fluid was transferred to the LT-α test card (Model S02A, Guangdong Xinda Biomedical Co., Ltd., China). The LT-α test reagent was applied to the designated reagent area of the test card. The subject’s name and sampling time were recorded on the test card, followed by a 10-min incubation period at room temperature. The test card was inserted into the quantitative analyzer (Model S03A, Guangdong Xinda Biomedical Co., Ltd.) for automated analysis. The analyzer provided qualitative results and quantitative LT-α concentration measurements in the tear fluid (Figure 1).

### Anterior segment optical coherence tomography (AS-OCT)

The examination was performed using the anterior segment optical coherence tomography (AS-OCT) system (CASIA2, Tomey Corporation, Japan). The examiner adjusted the refractive compensation parameters based on the subject’s refractive error. The focus knob and fixation target were fine-tuned until a clear corneal cross-sectional image and OCT scanning line/ring were visualized on the monitor. The corneal screening test mode was selected for automated quantitative analysis. Cross-sectional images of the cornea were acquired, and the thinnest corneal thickness was recorded (15).

### Corneal tomography

We recognize that corneal topography is the most widely used diagnostic method for keratoconus. Examinations were conducted using the Pentacam HR system (Oculus GmbH, Wetzlar, Germany; software versions 1.16r26 and 1.17r139). Corneal topographic maps were obtained for all subjects, and the following parameters were recorded: Holladay equivalent keratometry values for K1, K2, K_max_, and astigmatism (16).

### TMH and NI-BUT assessment

TMH and NI-BUT were evaluated using the OCULUS Keratograph 5 M Comprehensive Ocular Surface Analyzer (OCULUS Optikgeräte GmbH, Germany). Measurements were taken according to the manufacturer’s protocols, and the results were recorded for further analysis (17, 18).

### LLT measurement

LLT was assessed using the LipiView Ocular Surface Interferometer (TearScience, Inc., USA). During the examination, subjects were instructed to avoid rubbing their eyes to prevent the stimulation of the meibomian glands, which could alter lipid secretion and affect measurement accuracy. Proper head positioning and fixation were maintained to ensure consistent and reliable results (19).

### Meiboscore

Infrared imaging of the meibomian glands was performed using the OCULUS Keratograph 5 M instrument (OCULUS Optikgeräte GmbH, Germany). The upper and lower eyelids of each subject were imaged, and the images were analyzed for meibomian gland dysfunction. The degree of meibomian gland dysfunction was scored according to the percentage of gland loss. The Meiboscore grading criteria includes four levels: Score 0: minimal or no gland loss; Score 1: <33% of glands missing; Score 2: 33–66% of glands missing; Score 3: >66% of glands missing. The scores for the upper and lower eyelids of the same eye were summed to obtain a total MGD score for that eye, ranging from 0 to 6 points (20).

### Statistical analysis

Visual acuity measurements were converted into LogMAR units for statistical analysis. Continuous variables were expressed as mean ± standard deviation (mean ± SD). Categorical variables were expressed as quartiles. The Kolmogorov–Smirnov test was used to assess the normality of the data distribution. Pearson’s correlation analysis was used to evaluate the correlation between two sets of continuous variables. An independent samples *t*-test was used to compare two groups of continuous variables. An analysis of variance was used to compare three or more groups of continuous variables. *Post-hoc* pairwise comparisons were performed using the Bonferroni test if significant differences were found. Spearman’s correlation analysis was used to evaluate the correlation between two sets of non-normally distributed continuous or categorical variables. The Mann–Whitney U (rank-sum) test was used to compare two groups of non-parametric independent samples. Rank ANCOVA was used to analyze the influence of covariates on the dependent variable of non-normal distribution. The Kruskal–Wallis test was used to compare three or more groups of non-parametric independent samples. *Post-hoc* pairwise comparisons were performed using the Bonferroni test if significant differences were found. Pearson’s χ^2^ test was used to compare differences in gender distribution between groups. Differences were considered statistically significant at a *p*-value of < 0.05.

## Result

### Demographic characteristics of the HC and KC groups

No statistically significant differences were observed between the HC and KC groups in terms of age (*p* = 0.150) or gender (*p* = 0.467) distribution. Detailed demographic data are presented in Table 2.

**Table 2:** Demographic characteristics of the HC and KC groups.

Variable	HC Group	KC Group	*p*
Number of participants (*n*)	67	100	
Age (yrs)	29.78 ± 5.8	24.33 ± 5.4	0.150
Female/male (*n*%)	34/66	31/69	0.467
Clinical staging of keratoconus			
preclinical stage (*n*%)	/	30	
initial stage (*n*%)	/	17	
complete stage (*n*%)	/	53	

### Analysis of findings in the control and keratoconus groups

**Figure 2:**
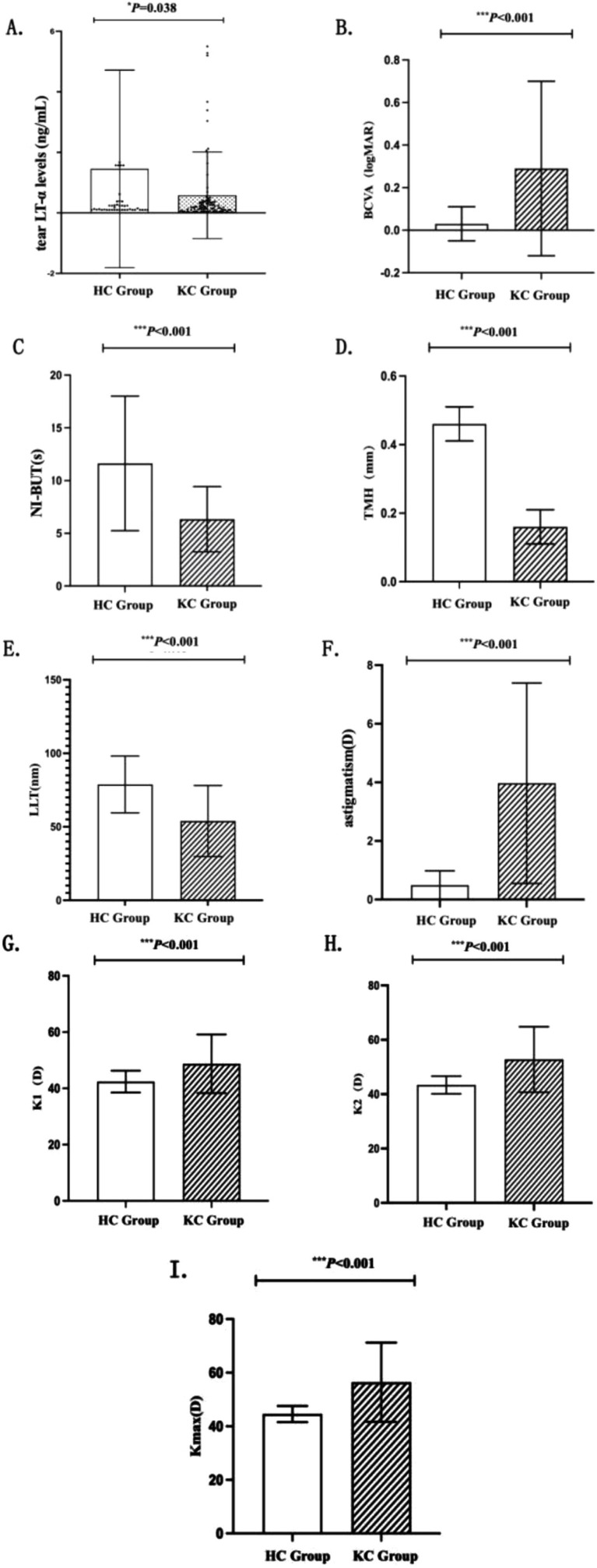
Histogram of the results of the HC and KC groups. Panels **(A–I)**: Compare HC vs. KC groups for: tear LT-α levels, BCVA, NI-BUT, TMH, LLT, astigmatism, K1, K2, and K_max_. Histograms show distributions and statistical differences. Error bars represent the standard deviation. Statistical significance is denoted as: *p < 0.05, **p < 0.01, and ***p < 0.001, with analyses performed using independent samples *t*-tests or Mann–Whitney U-tests appropriate for the data distribution.

A comparative analysis of ocular parameters between the KC and HC groups revealed the following findings (Table 3 and Figure 2): The HC group exhibited significantly higher tear LT-α levels than the KC group (*p* = 0.038). After adjusting for age using rank ANCOVA, the KC group showed significantly lower ranked tear LT-α levels compared to the HC group (*p* = 0.012). NI-BUT, TMH, and LLT were significantly lower in the KC group compared to the HC group (all *p* < 0.001). Meiboscore was significantly higher in the KC group compared to the HC group (*p* < 0.001). Significant differences in corneal topographic parameters, including K_max_, K1, K2, and astigmatism, were observed between the KC and HC groups (all *p* < 0.001).

**Table 3:** Findings in the control and keratoconus groups.

Variable	Research groups		*Z*	*p*
	HC group	KC group		
Tear LT-α levels (ng/mL)	1.57 ± 3.33	0.58 ± 1.43	−2.073	0.038
BCVA (logMAR)	0.03 ± 0.08	0.29 ± 0.41	−5.148	<0.001
NI-BUT (s)	11.63 ± 1.37	6.34 ± 3.10	−6.318	<0.001
TMH (mm)	0.46 ± 0.05	0.16 ± 0.05	−9.838	<0.001
LLT (nm)	78.80 ± 19.31	53.95 ± 24.15	−6.173	<0.001
Meiboscore	0 (0 ~ 1)	1 (0 ~ 1)	−3.895	<0.001
Astigmatism (D)	0.49 ± 0.49	3.97 ± 3.42	−7.295	<0.001
K1 (D)	42.41 ± 3.89	48.73 ± 10.41	−4.039	<0.001
K2 (D)	43.39 ± 3.27	52.77 ± 12.04	−6.722	<0.001
K_max_ (D)	44.58 ± 3.02	56.44 ± 14.79	−6.172	<0.001

Z, Mann–Whitney U-test statistic (for non-parametric comparisons).

### Data analysis of examination results among KC groups by clinical stage

**Figure 3:**
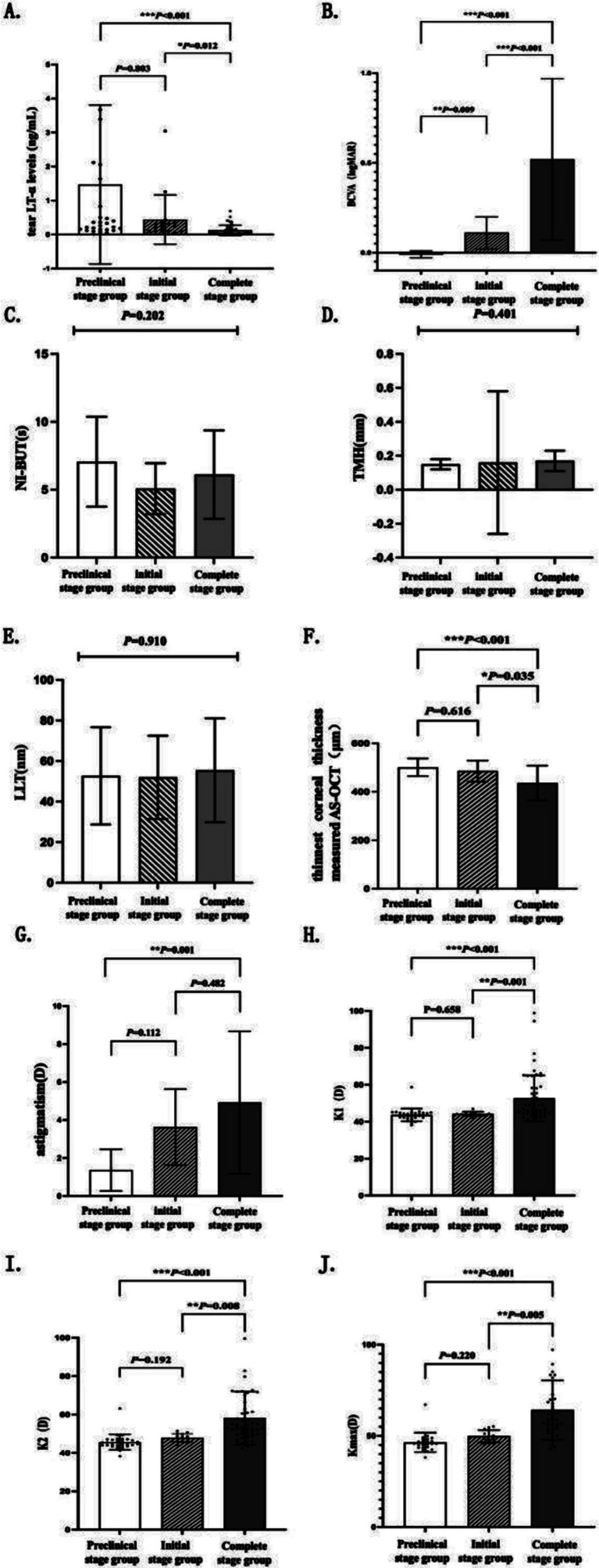
Histograms of examination results across clinical stages of keratoconus. Histograms **(A–J)** compare tear LT-α levels, BCVA, corneal thickness, NI-BUT, TMH, LLT, astigmatism, K1, K2, and K_max_ across the preclinical, initial, and complete keratoconus stages. Histograms illustrate the distribution and statistical differences of these parameters across the three clinical phases of keratoconus. Error bars represent the standard deviation. Statistical significance is denoted as: *p < 0.05, **p < 0.01, and ***p < 0.001, with analyses performed using analysis of variance or Kruskal–Wallis tests as appropriate for the data distribution.

The examination results for the preclinical, initial, and complete stages of keratoconus were analyzed and are presented in Table 4 and Figure 3. The tear LT-α levels in the latent, incipient, and advanced stages were (1.47 ± 2.34) ng/mL, (0.44 ± 0.73) ng/mL, and (0.13 ± 0.15) ng/mL, respectively. A significant difference was observed among the three groups (*H* = 26.840, *p* < 0.001), with tear LT-α levels progressively decreasing from the preclinical to the complete stage. Statistically significant differences were observed among the three groups in the following parameters (all *p* < 0.001): BCVA, thinnest corneal thickness measured by AS-OCT, corneal topographic parameters (astigmatism, K1, K2, and K_max_). No significant differences were observed among the three groups in NI-BUT, TMH, or LLT. A statistically significant difference was observed in the meiboscore among the three groups (*p* = 0.018).

**Table 4:** Data analysis of examination results among three groups.

Variable	Preclinical stage group	Initial stage group	Complete stage group	*H/F*	*p*
Tear LT-α levels (ng/mL)	1.47 ± 2.34	0.44 ± 0.73	0.13 ± 0.15	26.840	<0.001^bc^
BCVA (logMAR)	−0.01 ± 0.02	0.11 ± 0.09	0.52 ± 0.45	77.594	<0.001^abc^
NI-BUT (s)	7.06 ± 3.31	5.08 ± 1.87	6.11 ± 3.26	1.681	0.202
TMH (mm)	0.15 ± 0.03	0.16 ± 0.42	0.17 ± 0.06	1.827	0.401
LLT (nm)	52.70 ± 23.98	52.00 ± 20.50	55.46 ± 25.63	0.190	0.910
Meiboscore	1 (1 ~ 2)	1 (1 ~ 2)	1 (0 ~ 1)	8.060	0.018
Thinnest corneal thickness measured by AS-OCT (μm)	501.17 ± 36.43	485.52 ± 43.61	436.10 ± 71.35	23.262	<0.001^bc^
Astigmatism (D)	1.36 ± 1.10	3.63 ± 2.00	4.92 ± 3.75	12.799	0.002^c^
K1 (D)	43.64 ± 3.40	44.01 ± 1.26	52.68 ± 12.45	31.709	<0.001^bc^
K2 (D)	45.60 ± 4.00	47.89 ± 2.19	58.11 ± 13.86	44.980	<0.001^bc^
K_max_ (D)	46.36 ± 5.23	49.83 ± 3.32	64.16 ± 16.25	38.662	<0.001^bc^

Statistical significance annotations: a: preclinical vs. initial stage; b: initial vs. complete stage; and c: preclinical vs. complete stage. These annotations indicate the multiple comparisons between clinical stagings of keratoconus. H/F: Kruskal–Wallis H-statistic (non-parametric) or ANOVA F-statistic (parametric).

### Correlation between tear LT-α levels and ocular parameters in the HC and KC groups

In the HC Group: No significant correlation was observed between tear LT-α levels and the following parameters: BCVA (*p* = 0.200), NI-BUT (*p* = 0.601), TMH (*p* = 0.500), LLT (*p* = 0.607), and meiboscore (*p* = 0.432). Similarly, no significant correlation was observed with corneal topographic parameters, including astigmatism (*p* = 0.156), K1 (*p* = 0.141), K2 (*p* = 0.895), and K_max_ (*p* = 0.586).

**Figure 4:**
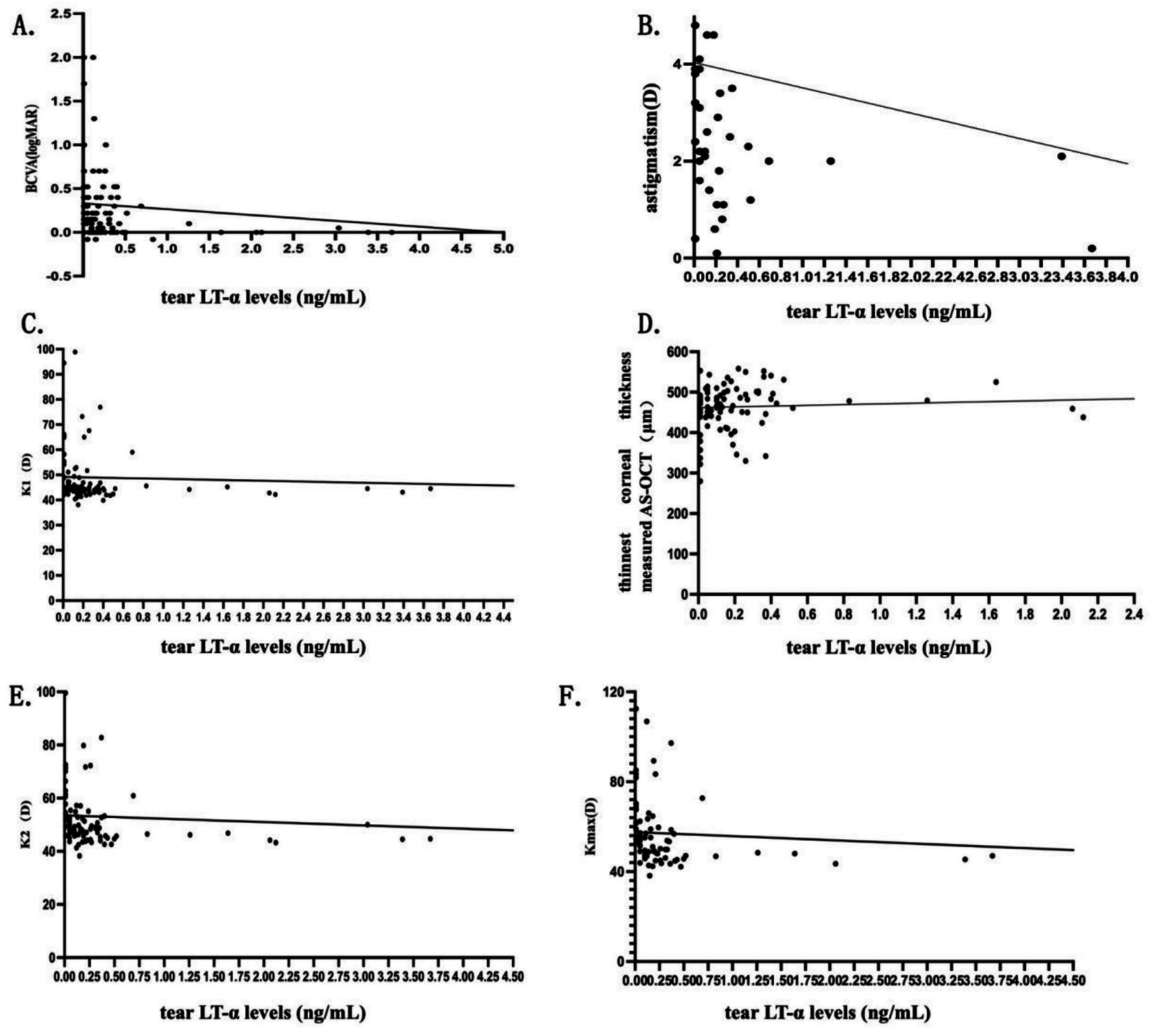
Scatterplots of correlation analysis in the KC group. Scatterplots **(A–F)** show correlations between tear LT-α levels and BCVA, corneal thickness (AS-OCT), astigmatism, K1, K2, and K_max_ in KC patients. The scatterplots visually represent the strength and direction of the correlations, with regression lines indicating trends.

In the KC Group, tear LT-α levels were positively correlated with the thinnest corneal thickness measured by AS-OCT (*p* < 0.001), and tear LT-α levels were negatively correlated with *p* < 0.001: BCVA, astigmatism, K1, K2, and K_max_. No significant correlations were found between tear LT-α levels and NI-BUT, TMH, LLT, and meiboscore (all *p >* 0.05). For detailed numerical data and graphical representations, refer to Table 5 and Figure 4.

**Table 5:** Correlation between tear LT-alpha levels and various findings in the KC group.

Inspection projects	*r_s_/r*	*p*
BCVA	−0.478	<0.001
NI-BUT	0.311	0.065
TMH	−0.077	0.453
LLT	0.001	0.989
meiboscore	0.142	0.116
thinnest corneal thickness measured by AS-OCT	0.341	<0.001
astigmatism	−0.377	0.006
K1	−0.341	<0.001
K2	−0.468	<0.001
K_max_	−0.495	<0.001

r_s_/r: Spearman’s rank correlation coefficient (for non-normal data) or Pearson’s r (for normal data).

## Discussion

In recent years, an increasing number of clinical studies have provided evidence supporting the involvement of inflammatory mechanisms in the pathogenesis of keratoconus (21). Jonescu et al. demonstrated the critical involvement of inflammatory responses in the development of keratoconus (22). Alterations in various inflammatory mediators, including tumor necrosis factor-alpha (TNF-*α*), interleukins (IL-1α/β, IL-6, IL-8, IL-17, IL-4, and IL-13), interferon-gamma (IFN-γ), and immunoglobulin E (IgE), have been found in the corneal tissues, tear film, and aqueous humor of keratoconus patients (3, 23–27). It is notable that elevated levels of matrix metalloproteinase-9 (MMP-9) have been observed in the tears (28), corneal tissues (29), and serum (30) of keratoconus patients. The upregulation of protease expression in response to these inflammatory factors increased the efficiency of collagen fiber degradation (31). This process led to an abnormal distribution and reduction in the number of stromal collagen lamellae, ultimately impairing the biomechanical properties of the corneal tissue and destroying its structural integrity, thereby contributing to the progression of keratoconus (32–34).

LT-α is essential for maintaining ocular surface immune homeostasis via the LT-α-TNFR2-Treg axis, contributing to tissue repair, goblet cell differentiation, and proper mucin secretion (35). Studies have shown that, in dry eye disease (DED) patients, tear LT-α levels are significantly reduced in most cases, reflecting impaired tissue repair mechanisms (36). Conversely, elevated LT-α levels in some patients point to immune overactivation and associated tissue damage (37). Recently, LT-α has gained attention as a promising biomarker for the diagnosis of dry eye disease (38).

While the specific role of LT-α in the onset and progression of keratoconus remains unclear, inflammation has been recognized as a key factor in its pathogenesis. This suggests that LT-α could potentially play a role in the disease mechanism. Supporting this finding, earlier research has identified notable changes in immune-related markers in the tears of individuals with keratoconus (39). For example, concentrations of IL-6, IL-8, IL-1β, and TNF-*α* are markedly elevated in keratoconus patients relative to healthy controls (40, 41). Furthermore, lymphocyte levels have been decreased in the peripheral blood of those with keratoconus.

The introduction of commercially available tear test strips has enabled rapid and precise measurement of tear fluid biomarkers in clinical practice. This technological progress has supported the present study in exploring the potential role of LT-α in the context of keratoconus.

The study uncovered several critical insights into tear LT-α levels in keratoconus patients. Compared to healthy controls, keratoconus patients exhibited markedly lower tear LT-α levels, with a progressive decrease observed across clinical stages: preclinical stage > initial stage > completed stage. The consistency between unadjusted and age-adjusted results, where *p*-values remained significant, reinforces the fact that group differences are independent of age effects. While no significant correlations were observed between tear LT-α levels and any examined parameters in the HC group, significant correlations were identified in the KC group. Specifically, tear LT-α levels were associated with BCVA and corneal topographic parameters, including K1, K2, K_max_, and astigmatism. These results imply that LT-α could play a role in regulating keratoconus development, potentially influencing disease progression and corneal structural alterations.

Our findings of reduced tear LT-α in KC may reflect multiple mechanisms: (1) impaired immune homeostasis, as LT-α is crucial for lymphoid tissue development and T-cell regulation, with its deficiency potentially disrupting corneal repair; (2) compensatory downregulation due to chronic inflammation, similar to observations in dry eye disease; and (3) broader immune dysfunction, consistent with lymphocyte depletion in KC.

Given that tear LT-α levels are reduced in most DED patients and that DED is a recognized risk factor for keratoconus, this study also analyzed the relationship between tear LT-α levels and tear film parameters in both groups. The results showed that the HC group had significantly lower values for NI-BUT, TMH, and LLT compared to the KC group, with statistically significant differences between the two groups. These findings are consistent with previous studies by Cingu et al. (2). The observed differences in tear film parameters between the groups may be attributed to several factors (42). Abnormal corneal morphology in keratoconus, such as corneal thinning and protrusion, may increase mechanical friction or disrupt tear film distribution (2, 43). Furthermore, reduced density and structural abnormalities of subbasal and stromal nerves in keratoconus patients can lead to decreased corneal sensitivity, ocular surface dryness, and tear film hyperosmolarity (43, 44). These sensory disturbances reduce afferent signaling to the central nervous system, potentially resulting in decreased tear secretion and further exacerbating tear film instability. It is notable that no significant correlation was found between tear LT-α levels and tear film parameters in the KC group. However, tear LT-α levels were associated with the severity of keratoconus. This finding suggests that the previously established view of DED as a risk factor for keratoconus may not be entirely accurate. Instead, only DED associated with immune dysregulation or hyperactivation may represent the true risk factor for keratoconus. Further research is necessary to confirm this hypothesis and clarify the underlying mechanisms.

This study underscores the growing importance of POCT in clinical practice. Unlike conventional laboratory tests, POCT provides unique benefits such as cost efficiency, exceptional sensitivity and specificity, user-friendly operation, and quick results. Currently, allergen-specific IgE testing has gained widespread use in clinical settings. The portability and rapid turnaround of POCT make it feasible for integration into routine keratoconus monitoring, particularly in resource-limited settings. The scope of POCT is anticipated to broaden, incorporating additional biomarkers such as matrix metalloproteinases (MMPs). Tests such as the MMP-9 reagent assay may soon become essential tools in clinical diagnostics, potentially improving the precision and effectiveness of diagnosing and managing a range of ocular and systemic disorders.

This study has several limitations. First, the enrolled participants were relatively older, which may limit the generalizability of the findings. Second, as a cross-sectional study, it did not monitor fluctuations in tear LT-α levels during the progression of keratoconus, making it impossible to determine whether tear LT-α levels can predict the risk of disease progression. Future studies should investigate changes in tear LT-α levels as the disease progresses and assess LT-α levels when disease progression halts.

Although this study analyzed the correlation between tear LT-α levels and tear film parameters, it did not include a separate DED group for comparison. Additionally, tear samples were collected at non-uniform time points, and the potential influence of diurnal variations on LT-α expression levels remains unclear, which may introduce data bias. Furthermore, this study did not measure other biochemical factors potentially involved in the pathogenesis of keratoconus, such as IL-6 and MMP-9. The sources and activities of these factors were also not analyzed. The study did not incorporate additional tear fluid testing methods, such as enzyme-linked immunosorbent assay (ELISA) or polymerase chain reaction (PCR), to examine critical factors such as TNF-*α* and IL-17A. Therefore, the potential interactions between these factors and LT-α in the pathogenesis of keratoconus cannot be ruled out.

Future research should focus on investigating the correlation between tear LT-α levels and other tear fluid biomarkers in keratoconus patients, evaluating tear protein markers in individuals with varying LT-α expression levels, and analyzing the relationship between these markers and clinical manifestations of keratoconus, including corneal topographic findings.

## Conclusion

In summary, this study analyzed tear LT-α levels, tear film parameters, and corneal topographic findings in both healthy controls and keratoconus patients, as well as across different clinical stages of keratoconus. The results demonstrated a significant correlation between tear LT-α levels and the severity of keratoconus. Tear LT-α levels can reflect the immune status of the ocular surface to some extent, further supporting the hypothesis that immune dysregulation may play a role in the pathogenesis of keratoconus. In clinical practice, rapid tear fluid testing systems represent a simple and efficient method for detecting tear fluid biomarkers. Further exploration of their application value in various ocular surface and corneal diseases is warranted to enhance diagnostic and therapeutic approaches.

## Data Availability

The original contributions presented in the study are included in the article/supplementary material, further inquiries can be directed to the corresponding author.
